# Supplementary material for: Nonreciprocal interactions give rise to fast cilium synchronization in finite systems
Source: Proc Natl Acad Sci U S A. 2023 Sep 27;120(40):e2307279120. doi: 10.1073/pnas.2307279120 (PMC10556628; doi:10.1073/pnas.2307279120)
Supplement: Supplementary file 1 — Appendix 01 (PDF) [file pnas.2307279120.sapp.pdf]

PNAS

Supporting Information for

Nonreciprocal interactions give rise to fast cilium synchronisation in finite systems

David J. Hickey, Ramin Golestanian and Andrej Vilfan

E-mail: ramin.golestanian@ds.mpg.de, andrej.vilfan@ds.mpg.de

This PDF file includes:

Figs. S1 to S2
Legends for Movies S1 to S2

Other supporting materials for this manuscript include the following:

Movies S1 to S2

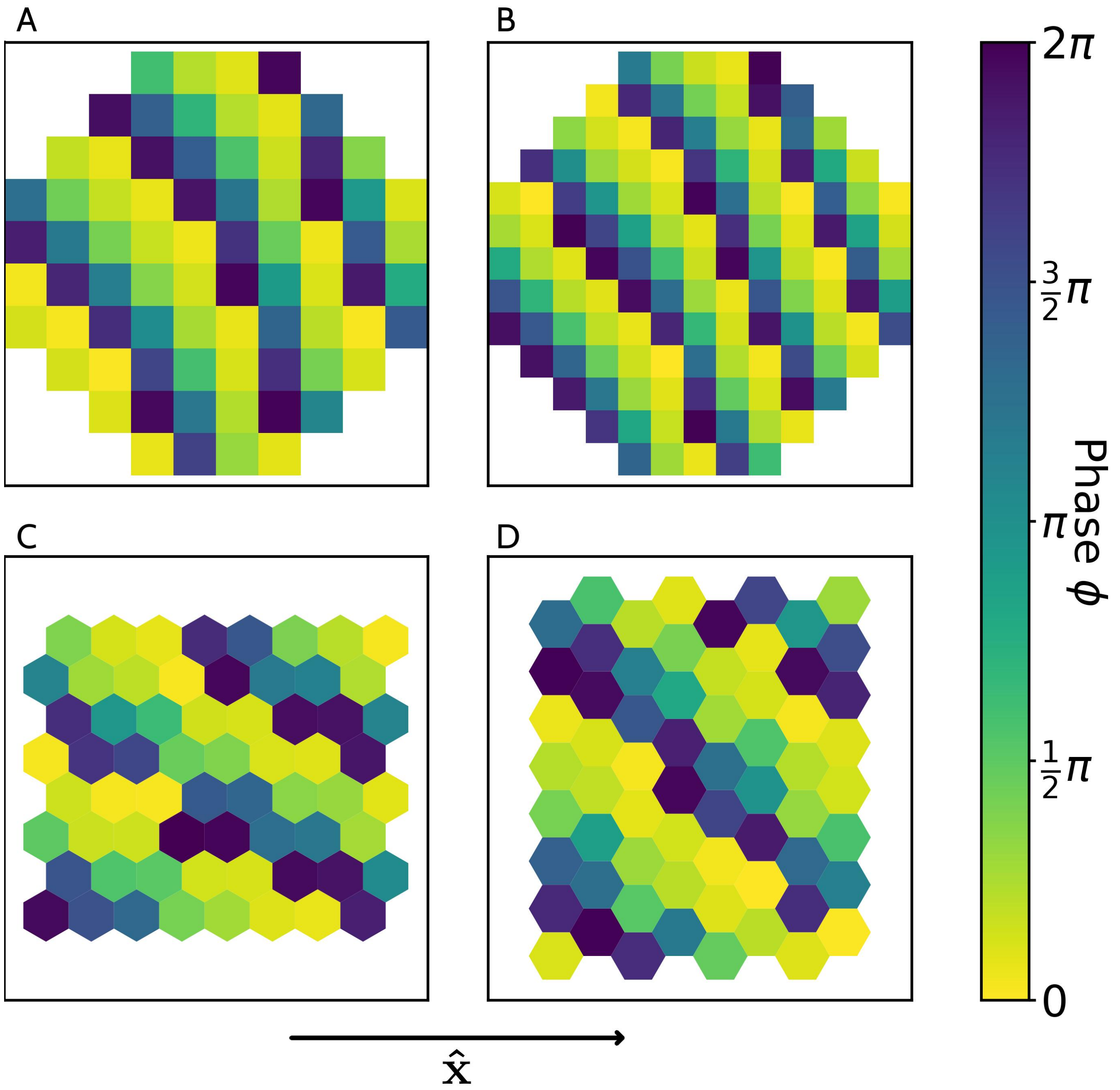

Fig. S1. Emergent metachronal waves on different lattices, with pumping direction in the x-direction (indicated). (A) 64 cilia arranged on a square lattice forming an octagon. (B) 144 cilia in the same arrangement. (C) 64 cilia on a hexagonal lattice, oriented such that one base vector is aligned with the direction of the power stroke. (D) as in (C), but with a lattice that is rotated by 90°.

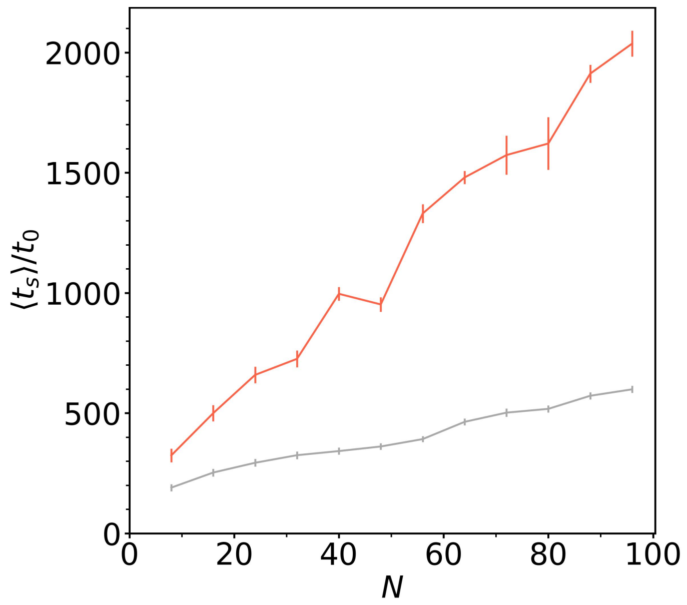

Fig. S2. Synchronisation times on a 1D lattice. The orange line shows cilia arranged along the y-axis (i.e. in the direction of weaker nonreciprocity). The grey line shows cilia arranged along the x-axis, as in Fig. 2C (main text). Synchronisation times still scale linearly when cilia are arranged in the y-direction, but are higher by some numerical factor, as expected from the weaker (but still present) nonreciprocity.

Movie S1. Animation of a metachronal wave in 2D.

Movie S2. Emergence of a metachronal wave on a lattice of 8×8 cilia.
